# Characterization of language abilities and semantic networks in very preterm children at school-age

**DOI:** 10.1371/journal.pone.0317535

**Published:** 2025-01-29

**Authors:** Marion Décaillet, Alexander P. Christensen, Laureline Besuchet, Cléo Huguenin-Virchaux, Céline J. Fischer Fumeaux, Solange Denervaud, Juliane Schneider

**Affiliations:** 1 Clinic of Neonatology, Department of Mother-Woman-Child, Lausanne University Hospital and University of Lausanne, Lausanne, Switzerland; 2 Department of Radiology, Lausanne University Hospital and University of Lausanne, Lausanne, Switzerland; 3 The Sense, Innovation, and Research Center, Lausanne, Switzerland; 4 Psychology and Human Development, Vanderbilt University, Nashville, TN, United States of America; 5 CIBM Center for Biomedical Imaging, Lausanne, Switzerland; 6 MRI Animal Imaging and Technology, Polytechnical School of Lausanne, Swiss Federal Institute of Technology Lausanne (EPFL), Lausanne, Switzerland; Education University of Hong Kong, HONG KONG

## Abstract

It has been widely assessed that very preterm children (<32 weeks gestational age) present language and memory impairments compared with full-term children. However, differences in their underlying semantic memory structure have not been studied yet. Nevertheless, the way concepts are learned and organized across development relates to children’s capacities in retrieving and using information later. Therefore, the semantic memory organization could underlie several cognitive deficits existing in very preterm children. Computational mathematical models offer the possibility to characterize semantic networks through three coefficients calculated on spoken language: average shortest path length (i.e., distance between concepts), clustering (i.e., local interconnectivity), and modularity (i.e., compartmentalization into small sub-networks). Here we assessed these coefficients in 38 very preterm schoolchildren (aged 8–10 years) compared with 38 full-term schoolchildren (aged 7–10 years) based on a verbal fluency task. Using semantic network analysis, very preterm children showed a longer distance between concepts and a lower interconnectivity at a local level than full-term children. In addition, we found a trend for a higher modularity at a global in very preterm children compared with full-term children. These findings provide preliminary evidence that very preterm children demonstrate subtle impairments in the organization of their semantic network, encouraging the adaptation of the support and education they receive.

## Introduction

Very preterm births (i.e., before 32 gestational weeks) are known to cause various adverse neurodevelopmental outcomes [1, 2]. Between 3–5 years, very preterm children present more difficulties in multiple cognitive domains compared with full-term children [3, 4]. Among these domains, language is often reported as problematic [5], with long-lasting impairments commonly reported in preterm children (for reviews see for example [6, 7]). With the exception of those without severe neonatal medical complications [8], language impairments seem to affect many subdomains. While early childhood delays are caught up in low-level language abilities (i.e., phonological processing and sight word reading) by early adolescence, high-level abilities (i.e., syntax, semantics, phonemic decoding, and verbal language memory) remain more often impaired in very preterm adolescents [9, 10]. Consequently, they need about eight times more academic support in writing and reading compared with their full-term peers [5]. One aspect of these long-lasting impairments concerns their vocabulary. Where Kunnari et al. [11] found no differences in expressive vocabulary for toddlers aged 2, Luu et al. [5] observed poorer receptive and expressive vocabulary at later ages in preterm children compared with full-term controls aged 8 and 12. However, later at the age of 16, they found that the deficit in receptive vocabulary was no longer present [9]. Moreover, while 3-year-old very preterm children uttered the same number of questions and negations, and had comparable lexical variability (i.e., number of nouns, verbs, adjectives, prepositions) compared with full -term children, they produced less varied sentence structure with, in addition, shorter and fewer words as well as fewer words roots [12]. Additionally, preterm children also exhibit impaired reading skills and comprehension as well as delayed grammatical development (e.g., [13]). These skills rely in particular on word comprehension [6]. Altogether these quantitative measures show that preterm children have poorer receptive and expressive language, semantics, and receptive grammar, reflecting a lower overall comprehension capacity (e.g., sentences, words, structure, verbal information) [14].

Moreover, most studies report that these impairments also affect verbal memory. At 3.5 years old, preterm children already present poorer phonological working memory (i.e., memory of words and non-words) than full-term children. Omizzolo et al. [15] discovered that despite preterm children having good long-delay verbal recall, their short-delay verbal recall remains impaired at 7 years compared with full-term controls. This persists during adolescence, with 9-16-year-old preterm children showing weaker linguistic working memory relatively to full-term children of the same age. This is evidenced by their poorer performance on tasks assessing sentence recall and semantic relationships identification [16].

Whereas language abilities and verbal memory have been well studied in very preterm children, the underlying complexity of semantic organization has not been explored yet. The way concepts are learned and progressively organized in memory predicts various higher-order abilities such as memory, abstract thinking or memory [17, 18]. Consequently, language difficulties in very preterm schoolchildren may mirror the underlying semantic organization. Indeed, children reinforce and expand their knowledge in their early years. During their first 7 years, they acquire on average 800 root words per year and then learn 1,000 new words per year until the age of 12 [19]. Vocabulary and concepts are stored in the semantic memory network [20]. Alongside this acquisition, children also weave links between these words and gain a deeper understanding and meaning of concepts. This enables them to use abstract and conditional reasoning [21]. While gaining knowledge and expertise in a particular domain, semantic structure becomes more cohesive and associative, thus they have better access to specific knowledge [17]. Semantic memory structure is considered to be built and influenced by experience [22], but there is a gap in knowledge concerning children who have early developmental adversity such as very preterm birth.

Recently, some studies have begun to focus on semantics in the form of networks using methods based on mathematical models and computational research (e.g., [23]). Concepts are represented by nodes (i.e., basic units of the network) which are interconnected (i.e., represented by the edges) [24]. These associations are based on different similarities, creating a network. This latter is structured by three mechanisms, the modularity (i.e., Q, subnetworks creation), the average shortest path length (i.e., ASPL, moving from one node to others), and the clustering (i.e., CC, closeness, or isolation of the nodes). These parameters influence the way we perceive and retrieve concepts. For example, the more distant the concepts are, the more difficult it is to make associations between them [23].

By investigating semantic networks in very preterm children, we aimed to examine whether their verbal difficulties could be attributed to an underlying problem with the organization of the semantic memory, specifically in terms of the encoding and storage of concepts. One common and simple way to investigate semantic networks is the verbal fluency task where participants are asked to give as many words as possible in a specified category (e.g., animal, food, words beginning with a given letter) in a fixed time (usually 60 or 90 seconds). Given the language and memory difficulties present in the population of very preterm children, we expected to find differences in their semantic memory organization compared with full-term children. More specifically and based on the existing literature, we hypothesized (1) higher ASPL, (2) lower CC and (3) higher Q. Differences in their semantic memory structure may point to differences in learning and encoding. This could help to adapt current teaching and specialized educational support, as some pedagogical practices are known to foster a more flexible semantic network [25].

## Materials and methods

### Ethics

The present study is part of a larger project titled *Long-term impact of early nutritional and pain management in very preterm infants on brain health and function* and approved by the University ethical committee (CER-VD, protocol no. 2019–01056). All methods were carried out in accordance with the STROBE 2007 guidelines and with the principle of the Declaration of Helsinki. Each parent signed informed consent for their child, and oral consent from each child was obtained.

### Design of the study

The present study was nested in an observational cohort study of very preterm children who were recruited from birth at the Clinic of Neonatology of the University Hospital of Lausanne between 2011 and 2013. They were iteratively followed up at the Development Unit of the same hospital. Further details on this cohort study have been previously described [26].

For comparison, we used a reference group of full-term children of the same age. They were recruited by one of the authors in the French-speaking side of Switzerland between 2018 and 2021 as part of a parallel study about schooling and neurocognitive development [25].

### Participants

#### Very preterm children

The initial cohort comprised 51 very preterm neonates. Throughout the study, 2 (3.9%) patients died during the follow-up, 2 (3.9%) were excluded due to complex social situations, 2 (3.9%) were lost at previous appointments, 2 (3.9%) were unreachable and 2 (3.9%) refused to participate. Finally, 3 (5.9%) children were not able to perform the verbal fluency task (for details see S1 Fig).

Therefore, in total, 38 (75% of the initial cohort) very preterm children born between 25 and 31 gestational weeks and aged 8–10 years took part in the present study. They were tested separately in quiet rooms during their follow-up appointment at the Developmental Unit between October 2019 and October 2021.

#### Full-term children: Reference group

Thirty-eight full-term children aged 7–10 years were matched based on age and gender. They performed an animal verbal fluency task alongside other different tests during their participation in the parallel study [25] and were tested at the same hospital or at their school.

### Materials

#### Verbal fluency

The very preterm children performed the Word Generation task, a subtest of the NEPSY-II [27], a neuropsychological battery where children must generate words. It is divided into two subcategories, the *Semantic Word Generation* score, and the *Initial Letter Word Generation* score. In the Semantic Word Generation, children have sixty seconds to name all the words belonging to the category (i.e., animals and foods/drinks) they could think of. Then, in the Initial Letter Word Generation, they are given a letter (i.e., F and S) and have to produce all the words starting with this letter they could in sixty seconds. Words were immediately transcribed. We discarded non-animals, imaginary animals, and redundant plurals and offspring. The total number of correctly generated words was used. This subtest also comprises a comparison score calculated based on the scores of the two subcategories. It is aimed to compare fundamental processes (i.e., semantic) and more complex ones (i.e., initial letter).

The full-term children did not complete the whole NEPSY but only performed a verbal fluency task about animals.

Based on previous research [25, 28], we selected the animal category to compute the semantic networks and compare the two groups.

#### Socioeconomic status (SES)

Regarding the very preterm children, SES was assessed through the Largo [29]. It is composed of two scores, the mother’s education (or current work if applicable), and the father’s current job, both scoring from 1 to 6 (1 representing higher SES). Full-term children’s SES was determined by both parent’s educational level and current job (both scoring from 1 to 4, 1 representing lower SES). These scores were summed and averaged between both parents. To compute the comparison between both groups, the very preterm score was reversed, and all scores were set as a percentage.

#### Non-verbal intelligence

The *Matrix Reasoning* subtest from the Wechsler Intelligence Scale for Children 5^th^ Edition (WISC-V) [30] was used to measure the intelligence of very preterm children. They were asked to choose the missing piece that completed the sequence of coloured visual pattern matrices. Full-term children filled the black and white Raven’s Progressive Matrices [31]. They were also asked to select the missing piece of black and white visual pattern matrices. The two scores were converted in success rates by dividing the number of correct responses by the maximum possible score and multiplying this division by one hundred.

### Data analyses

#### Word Generation

All three scores (i.e., Semantic Word Generation, Initial Letter Word Generation, and Comparison scores) are expressed as standard scores ranging from 1 to 19 and have been transformed into standard IQ scores for conventional purposes. Comparison score is calculated based on Semantic Word Generation and Initial Letter Word Generation standard scores. Lower Comparison scores are representative of a profile with good capacity for vocabulary production and less effective strategies for retrieving non-categorical information, while the highest scores are the opposite.

#### Group comparison

The socioeconomic background and intelligence having a significant impact on verbal abilities, both variables were tested between groups using t-tests to assess group comparability. We then used them as covariates in two adjusted semantic network analyses (i.e., a first adjusted model with SES as covariate and a second adjusted model with SES and non-verbal intelligence as covariates).

To ensure the validity of the use of the *Matrix Reasoning* subtest success rate for very preterm children, a Pearson correlation was made between success rates and the *Full Scale IQ* T-scores from the WISC-V. We found a strong correlation between both scores (r (36) = 0.729, p < .001) allowing us to use the success rate as a measure of non-verbal intelligence. We kept the Matrix Reasoning subtest to be as close as possible to the Matrix task performed by the full-term children.

#### Semantic network analysis

The semantic network analysis was computed on the animal category. The network is decomposed in nodes and edges. Each node represents a category exemplar (i.e., in the present study an animal). Edges are the associations between two category exemplars. They represent the capacity of the groups to generate the second exemplar when they have produced the first one (e.g., generate both *lion* and *tiger*). The edges were constructed as undirected (i.e., no arrows or bidirectional relationships) and weighted (i.e., cosine similarity). However, in the network measures, the constructed networks were treated as undirected and unweighted.

The fluency data were preprocessed and semantic network components were measured using the public-available SemNA pipeline in R [32] using the R packages SemNetCleaner (version 1.3.4), SemNetDictionaries (version 0.2.0), and SemNet (version 1.4.4) [32], NetworkToolbox (version 1.4.2) [33], readxl (version 1.4.3) [34], RDS (version 0.9.10) [35].

*Network estimation*. The data were first pre-processed and cleaned with the SemNetDictionaries [36] and SemNetCleaner [37] tools. Repeated answers, non-categorical (i.e., others than animals), and fictional animals were excluded. Misspelled words and root variations (e.g., plural or adults and their offspring) were corrected and changed to their root (respectively, e.g., cats → cat). A binary response matrix was constructed with these cleaned responses. Its columns represent all the different exemplars given by the sample and its rows represent the participants. This matrix is filled out by 1 (if an exemplar was generated by that participant) and 0 (if that exemplar was not).

To compare the vocabulary size and the variety of words of the two groups, an independent t-test was run on the average total number of these cleaned responses given by each child and a chi-squared test on the total number of unique cleaned responses of both groups (i.e., all different words generated by at least one participant in each group). Network estimation can be adapted based on these results.

Then, we used a bootstrapping approach to simulate and compare semantic networks of both groups. We applied the case-wise bootstrap method, meaning that for each group, we resampled with replacement from the participants in their respective groups. A 1000-iterative procedure was conducted, entailing the computation of 1000 bootstraps. Each bootstrap sample has as many participants as the original sample but with some participants being included more than once and others not being included at all. This approach creates replicate samples with similar properties to the original samples, therefore, by using bootstrap with replacement, we test against groups that are assumed to be generally representative of each population. Furthermore, to preclude the possibility that observed differences in network structure were attributable to differences in vocabulary size, we used the number of cleaned response and the number of edges of each child as covariates. Consequently, in each bootstrap sample, we resampled the same participants’ covariate measures. Additionally, for the two adjusted models, the SES and non-verbal intelligence were included as covariates. The bootstrap handled finalizing (i.e., we used only responses given by at least 2 participants) and equating (i.e., networks of both groups in each sample are compared using the same nodes [38]) with each replicate sample of nodes. Equating the number of nodes aims to reduce the confounding effect of number of nodes to solely focused on their differences in connectivity. We used the similarity to avoid negative associations between nodes in the network (i.e., all values are positive ranging from 0 (two responses do not co-occur) to 1 (two responses always co-occur)). In addition, we applied the Triangulated Maximally Filtered Graph (TMFG; [39]) as a filtering method on the words association matrix. It minimizes the noise and potential spurious associations by constructing a sub-network to capture only the most relevant information (i.e., removal of spurious associations and keeping the highest correlations) within the original network. This approach constructs a sub-network by starting with the initial tetrahedron which consists of four nodes with the highest sum of correlations to all other nodes. Then it iteratively adds a new node which is connected to three existing nodes to create a triangulated graph that retains the strongest connections while remaining planar. This iterative process will continue unless all nodes have been integrated to the network (for more details, see [39]). Additionally, the TMFG method keeps the number of edges the same between groups, therefore newly created networks will be comparable since they will have the same number of nodes and edges. Thus, this eliminates the possibility of confounding different network structures owing to a difference in the number of edges [40, 41]. At the end of this 1000-iterative procedure, the network of the two groups was estimated.

**Fig 1:**
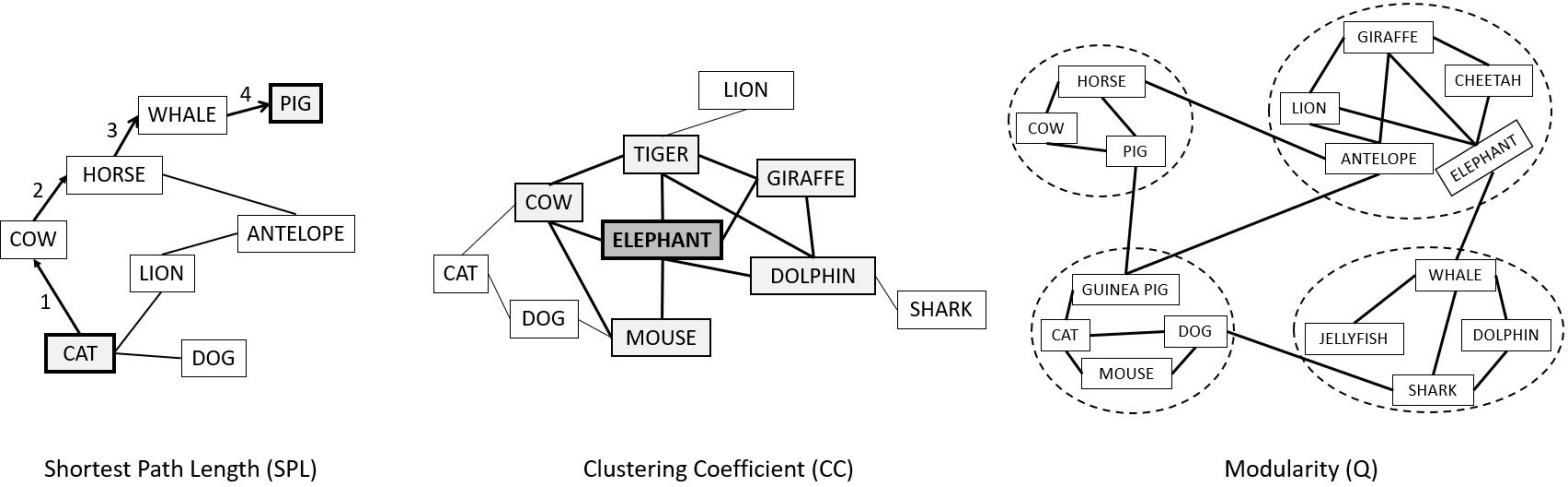
Visual representation of the three semantic coefficients for the animal category, -i.e., the Shortest Path Length (SPL), the Clustering Coefficient (CC) and the Modularity (Q). The ASPL cannot be visually represented but is the average of all SPL of the network. Note. Adapted from “Structural differences of the semantic network in adolescents with intellectual disability”, by K. Nilsson, L. Palmqvist, M. Ivarsson, A. Danielsson, M. Annell, D. Schöld, & M. Socher, 2021, *Big Data and Cognitive Computing*, 5(2), p.2 (https://doi.org/10.3390/bdcc5020025) with permission. Copyright 2021 by the authors.

*Network analyses*. Based on these estimated networks, we computed the 3 network coefficients for both groups (see Fig 1 for visualization). The Average Shortest Path Length (ASPL) refers to the average shortest number of steps (i.e., edges) we need to get between any pair of nodes in the network. It measures the global organization. Higher ASPL means that the semantic network is more spread out (i.e., less interconnected) and that people are less likely to make associations due to greater distances between associations [42]. And lower ASPL represents a more interconnected network with shorter distances between associations. Thus, lower ASPL increases the possibility of reaching more distant associations faster, like in creativity [23, 43]. The Clustering Coefficient (CC) refers to the extent that two neighbours of a node will be neighbours themselves. It illustrates the manner in which the semantic network is organized at a local level [32]. The higher this coefficient is, the more likely it is that the nodes close to each other will be connected, resulting in a more interconnected network [23]. The third coefficient, the Modularity Coefficient (Q) represents the extent to which the network can be compartmentalized into small sub-networks. A sub-network can represent an animal category (e.g., sea animals, savannah animals, farm animals, pets). The higher the coefficient, the more connections there are within a sub-network and the fewer there are with nodes in other subnetworks, making sub-networks more distinct from each other.

Then we first ran an unadjusted model and applied a t-test on the three networks coefficients (i.e., ASPL, CC, Q) to examine differences across both groups. Thanks to a specially adapted version of the code (i.e., adapted version of the pipeline from [32]), we then ran two adjusted models. We applied a first ANCOVA with the SES as a covariate and a second with the SES and the non-verbal intelligence as covariates. For all three models, the number of cleaned responses and edges were also used as covariates.

Bootstrapping small samples can result in an overrepresentation of outliers. Given the relatively small size of our groups, these latter could generate significant variation in our analyses. Therefore, the three 1000-iterative bootstrapping analyses were repeated twenty times. We reported the proportion of times ASPL, CC, and Q were significantly different between the two groups to demonstrate consistency in the results and that they were not the result of a single chance event.

As the visualization of the semantic network cannot be pooled over the twenty tests and cannot be computed with the addition of the covariates, bar plots of the coefficients average of the 20 tests for the unadjusted and the two adjusted models are provided in the *Supplementary Material* to compare the two groups.

## Results

### Demographics of very preterm children

Several perinatal factors associated with prematurity may have an impact on subsequent language and memory development (for details, see Table 1). At school-age, while some very-preterm children underwent speech therapy, they exhibited no hearing impairment, and their vision impairments were corrected with glasses. In addition, they were within age-normed standards regarding their general IQ and verbal comprehension (for further details, see Table 2).

**Table 1:** Neonatal characteristics.

Measures		Mean (s.d.)/number (%)	Range
**GA in weeks, M(s.d.)**		27.1 (1.48)	25–31
**Birth weight (grams), M(s.d.)**		908 (248)	517–1590
**Apgar 5min, M(s.d.)**		7(2)	3–10
**Multiplet, N(%)**		10 (26)	/
**Intraventricular hemorrhage, N(%)**			
	**Grade I**	7 (18)	/
	**Grade II**	3 (7)	/
	**Grade III**	1 (2)	/
	**Grade IV**	2 (5)	/
**Bronchopulmonary dysplasia, N(%)**			
	**Mild**	9 (23)	/
	**Moderate**	5 (13)	/
	**Severe**	6 (15)	/
**Retinopathy of Prematurity (ROP)** ^**a**^ **, N(%)**			
	**Grade I**	1 (2)	/
	**Grade II**	1 (2)	/

^a^ One patient was treated with laser, both had no sequalae at school-age.

**Table 2:** School-age characteristics.

Hearing Impairment, N(%)	0	/
Vision Impairment, N(%)		
Various pathologies corrected with glasses	11 (28)^b^	/
Strabismus	1 (2)	/
WISC V–Full Scale IQ, M(s.d.)	103.61 (11.31)	78–127
WISC V–Verbal Comprehension Index, M(s.d.)	97.84 (14.90)	69–135
Speech Therapy, N(%)	12 (31)	/

^b^ 10 children had a complete correction, and 1 child had no binocular vision.

### Word Generation of the very preterm children

Very preterm children performed within age-normed standards for the semantic score with a potential deficit for the initial letter score, with a score corresponding to the lower standards (see Table 3 for details). The lower comparison score (*M* = 84.73, *SD* = 11.18) indicated a good capacity for vocabulary production but less effective strategies for retrieving non-categorical information.

**Table 3:** Results of the Word Generation subtest in very preterm children.

	Very preterm Children		Norms
	Mean(s.d.)	Range	
**Semantic**	101.89 (2.54)	75–125	100 (15)
**Initial Letter**	85.81 (9.90)	70–105	100 (15)
**Comparison**	84.73 (11.18)	95–105	/

The scores are expressed as standard scores. There are no norms for the comparison score, but lower scores represent good capacities for vocabulary production with less effective strategies for retrieving non-categorical information, while the highest scores reflect the opposite pattern.

### Group comparison

Very preterm and full-term children demographics were assessed to establish their comparability (see Table 4 for details).

**Table 4:** Demographic and non-verbal intelligence comparison between very preterm and full-term groups.

	Group				
	Very preterm children	Full-term children	t-test or χ^2^	*p* value	Cohen’s d or φ
**N (% girls)**	38 (52)	38 (52)	0	1	0
**Age in years, M(s.d.)**	8.78 (0.458)	8.82 (0.715)	0.32	0.75	/
**Bilingualism, N(%)**	18 (47)	11 (29)^c^	2.46	0.17	0.18
**With mother tongue other than French, N(%)**	6(16)	2(5)	2.24	0.14	0.17
**Socioeconomic status, M(s.d.)**	58.6 (25.1)	59 (18.1)	0.07	0.94	0.02
**Non-verbal Intelligence, M(s.d.)**	51.8 (11.1)	88.7 (9.82)	73	< .001	/

Note that the SES was scored as a percentage, while the Non-verbal Intelligence was a success rate.

^c^ There were one missing data for this group and note that one of the children was a German speaker and completed the task in German.

### Semantic network analysis

#### Verbal fluency

Overall, very preterm children showed lower quantitative semantic abilities compared with full-term children (see Table 5 for details).

**Table 5:** Word Generation scores separated by group.

	Group				
	Very Preterm Children	Full-term Children	Welch’s t-test or χ^2^	*p* value	Cohen’s d or φ
**Mean total number of responses, M(s.d.)**	13.05 (3.48)	17.92 (5.16)	4.82	< .001	/
**Unique responses, N**	136/242	202/242	37.7	< .001	0.39
**Responses produced only by one child, N**	57	96	/	/	/
**Responses not provided by the other group, N**	40	106	/	/	/

#### Network coefficients—Unadjusted model

With the number of edges and cleaned response used as covariates, the twenty case-wise bootstrap network analyses demonstrated that despite small samples, very preterm children had a longer Average Shortest Path (M_*ASPL*,*VPT*_ = 3.378 vs M_*ASPL*,*FT*_ = 3.090) and a lower Clustering Coefficient (M_*CC*,*VPT*_ = 0.691 vs M_*CC*,*FT*_ = 0.706) than full-term children in every tests. Regarding the Modularity Coefficient, very preterm children showed a higher coefficient (M_*Q*,*VPT*_ = 0.604) than full-term children (M_*Q*,*FT*_ = 0.588) in 70% of the tests (i.e., 14 tests out of 20) (for the t-test results and the bar plots of the twenty case-wise bootstrap analyses, see the S1 File).

#### Network coefficients—Adjusted models

When the SES was added as a covariate, the twenty case-wise bootstrap network analyses also demonstrated that very preterm children presented a longer Average Shortest Path Length (and M_ASPL,VPT_ = 3.312 vs M_ASPL,FT_ = 3.168) and a lower Clustering Coefficient than full-term children (M_*CC*,*VPT*_ = 0.695 vs M_*CC*,*FT*_ = 0.702) in 19 tests out of 20. Regarding the Modularity Coefficient, very preterm children showed a trend for a higher coefficient (M_*Q*_ = 0.600) than full-term children (M_*Q*_ = 0.5932) with 55% of significant tests (i.e., 11 tests out of 20) (for the ANCOVA results and the bar plots of the twenty case-wise bootstrap analyses, see the S2 File).

When both SES and non-verbal intelligence were added as covariates, the analyses showed significant differences only regarding the Clustering Coefficient, very preterm children (M_*CC*_ = 0.695) having a lower Clustering Coefficient than full-term children (M_*CC*_ = 0.702), 14 times out of 20. Indeed, very preterm children (M_*ASPL*_ = 3.320) presented a trend for a longer Average Shortest Path Length than full-term children (M_*ASPL*_ = 3.158), with 55% of significant tests (11 tests out of 20). In contrast, the Modularity Coefficient, demonstrated no significant difference (only 8 times out of 20) between very preterm children (M_*Q*_ = 0.600) and full-term children (M_*Q*_ = 0.592) (for the ANCOVA results and the bar plots of the twenty case-wise bootstrap analyses, see the S3 File).

## Discussion

Schoolchildren born very preterm are known to present language weaknesses, measured through quantitative tools. However, qualitative aspects of language are less investigated. In the present study, we examined the organization of concepts in verbal memory of very preterm schoolchildren. Using an animal verbal fluency task, we depicted a section of their semantic network (i.e., the encoding and storage of concepts) and compared it with the semantic network of schoolchildren born at term, based on regular network metrics. Overall, very preterm children presented longer ASPL (i.e., distance between concepts) and lower CC (i.e., local interconnectivity) than full-term children. In addition, a trend for a higher Q (i.e., compartmentalization into small sub-networks) was observed in very preterm children. Here, we discuss this finding through the lens of known processing speed and language use in very preterm children and how pedagogical practices could be seen as a preventive action specifically targeting semantic organization alteration, with the aim of improving language abilities.

Very preterm schoolchildren are known to have language impairments, impacting semantics, expressive vocabulary, verbal language memory, and grammar [10, 13]. In the present cohort, despite overall good expressive and receptive language abilities as evidenced by age-normal performance on the *Word Generation* subtest of the NEPSY and the *Verbal Comprehension Index* of the WISC-V, 12 very preterm children required speech therapy. Moreover, they presented subtle difficulties in retrieving information when not organized into categories, with a consequently low comparison score at the *Word Generation* subtest. These difficulties may reflect either weaknesses in search strategies due to poorer working memory and inhibition capacity (i.e., executive functions) or a deficit in orthographic fluency or phonological awareness. In other words, semantic representations (i.e., lexical semantics) have been encoded and stored in long-term memory and very preterm children have access to them. However, they have not stored orthographic/phonological representations or have difficulty retrieving them. In addition, compared with the full-term children in the present study, the very preterm children generated fewer animal names, less unique ones, and presented a poorer diversity. Consequently, if we consider only quantitative measures, we might think that very premature children have a lack of vocabulary and therefore suggest educational support based on learning additional vocabulary. Yet, the underlying difficulties might lie elsewhere. Indeed, the semantic network of very preterm children was less interconnected at a global (ASPL) and a local (CC) level than observed in full-term children. Additionally, very preterm children show a tendency to organize their concepts into more subcategories (Q) and thus would have a more rigid organization than full-term children. Given that more distant and less embedded concepts will be harder to be retrieved and used concomitantly with other concepts [17], this qualitative perspective provides new understandings on their linguistic and cognitive difficulties, namely the way they organize and connect information. Establishing associations with distant concepts is more difficult, and it is harder to retrieve information when combined with a less organized network. Therefore, the reduced words production of very preterm children could be partly attributed to inadequate semantic organization. We can observe this difficulty in young childhood, at 18 months, as very preterm infants take more time to respond to spoken demands [44]. These authors linked this slowness to the amount of vocabulary the children had, regardless of their prematurity status. However, this phenomenon might also be associated to the semantic network organization and the difficulty encountered by children in accessing information. Across development, this may have a cascading effect on learning processes [45]. In fact, very preterm children have a lower processing speed in various tasks (e.g., [46]). This could reflect an inadequate semantic network organization with the difficulty to relate the information received to known concepts, as well as to retrieve and select the appropriate concepts to respond. Furthermore, while very preterm children show memory impairments in various domains (for a review see Anderson [47]), there is evidence that a less interconnected word will be less easily recalled during a memory cued recall task [48]. Therefore, their less organized network could be partly responsible for their neurodevelopmental deficits.

Very preterm children are known to experience multisensory difficulties [49, 50], that are responsible for higher-level cognitive impairments (for a review see Wallace et al. [51]) including language and memory [52]. Therefore, their inability to organise new concepts optimally might be partly explained by multisensory difficulties during the learning process. In view of this vulnerability, it might be appropriate to integrate interventions promoting multi-sensoriality into the learning process. Pedagogies using didactic materials engaging multiple senses, in active-based form, such as the Montessori pedagogy [53], were found to positively impact semantic networks’ structure. Indeed, Montessori schoolchildren compared with traditionally-schooled children, have a higher CC and lower ASPL and Q [25]. Therefore, it might be relevant to see pedagogy as a preventive measure for very preterm children. Providing access to educational settings and learning materials that reinforce and train complementary sensory channels will foster qualitative language abilities. Focusing more on meaning and integrating new words while engaging multiple senses rather than teaching isolated concepts, might allow children to build a more interconnected and organized network.

However, it should be noted that when both the SES and the non-verbal intelligence were added as covariates, very preterm children only demonstrated a lower CC and a trend for a higher ASPL. Given the importance of intelligence and SES on language development, differences in semantic processes may be partly underpinned by differences in non-verbal intelligence and SES, with the caveat that different measures of non-verbal intelligence were used for the two groups, which may have impacted the results.

Some limitations need to be acknowledged. First, the experimental settings for the very preterm and full-term children were not similar. For the former, data were collected in the framework of a routine clinical assessment in the context of a longitudinal study, while for the latter, data were collected in the framework of a voluntary-based experiment. Consequently, contexts may not be similarly stressful. Second, the very preterm children constituted a heterogeneous group (i.e., gestational age, birth weight, or speech therapy), which could have introduced some confounding effects. Future studies might want to test more homogeneous groups to better understand the specific implications of the different characteristics of very preterm children. Third, bootstrapping in small sample sizes being severely affected by outliers, given our modest sample-size, the analysis was not completely robust. And we recognise that the decision to perform 20 repetitions was subjective, given the absence of a precedent. Nevertheless, our results showed some consistency suggesting that although both groups were small, there did not appear to be issues related to outliers. Yet, it would be relevant to reproduce this study with a bigger cohort to confirm these results. Indeed, our results only showed a tendency towards lower modularity in very premature children, and the findings may be more conclusive with a larger cohort. Fourth, we only evaluated animal-related semantic network. It could be interesting to repeat this task with other semantic categories, such as food or hobbies. In fact, due to its advantages (e.g., generational, cultural, linguistically independent), the semantic category of animals is the most widely used [54]. But depending on the child’s interest in the category, the organization of the network may change. Fifth, we acknowledge the non-verbal intelligence measure was not the same for both groups, nevertheless, we took the most similar measure and attested to its validity for the very preterm children. However, although this allows us to exclude extreme cases and ensure intra-group homogeneity, it should be noted that the scoring is different with the Raven’s Progressive Matrices allowing children to go further compared to the subtest of the WISC-V, which could have biased the results with a tendency for higher scores for full-term children. Future studies might want to use the same measure to ascertain the impact of intelligence on semantic processes. Finally, we only have a cross-sectional perspective. As longitudinal studies show that the clustering coefficient diminishes with age in healthy population [55], it would be interesting to see if it is also the case with very preterm cohorts. In addition, given that very preterm children catch up with some of their weaknesses in adolescence (i.e., phonological processing, sight word reading and linguistic working memory, as mentioned above) [9, 16], we could investigate whether their semantic networks also normalize or follow the same pattern as those of full-term children while keeping these differences.

In conclusion, this study is one of the first to highlight qualitative aspects of language organization of very preterm children compared with full-term children, revealing subtle impairments. In fact, their semantic network appeared to be less connected, probably related to their processing speed and language difficulties. Given the importance of semantic network structure in higher cognitive functions, these findings encourage larger, longitudinal studies in this field. This will lead to improved support and teaching practices for very preterm children.

## Supporting information

S1 Fig Flowchart of the very preterm and full-term children. (TIF)

S1 File Results of the twenty case-wise bootstrap network analyses of the unadjusted model. (DOCX)

S2 File Results of the twenty case-wise bootstrap network analyses of the adjusted model for the SES. (DOCX)

S3 File Results of the twenty case-wise bootstrap network analyses of the adjusted model for the SES and intelligence. (DOCX)
