# Supplementary figures and images for: Characterization of language abilities and semantic networks in very preterm children at school-age
Source: PLoS One. 2025 Jan 29;20(1):e0317535. doi: 10.1371/journal.pone.0317535 (PMC12140111; doi:10.1371/journal.pone.0317535)

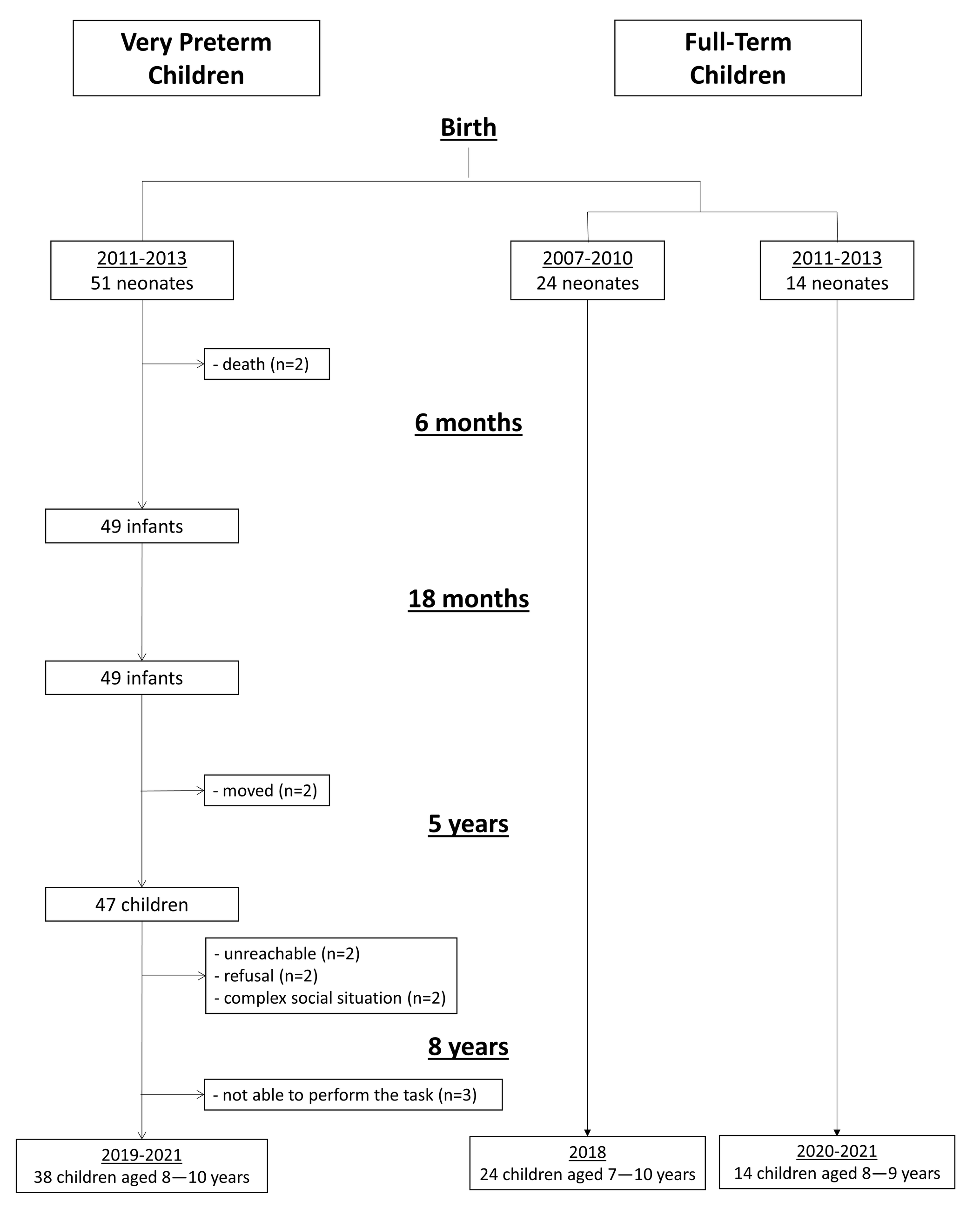

Supplement: S1 Fig — (TIF) [file pone.0317535.s001.tif]
