# Supplementary material for: Characterization of language abilities and semantic networks in very preterm children at school-age
Source: PLoS One. 2025 Jan 29;20(1):e0317535. doi: 10.1371/journal.pone.0317535 (PMC12140111; doi:10.1371/journal.pone.0317535)
Supplement: S1 File — (DOCX) [file pone.0317535.s002.docx]

**Unadjusted model**

**Results of the twenty case-wise bootstrap network analyses**

**Table 1. T-test results of the 1rst test**

|  | **FT** | **VPT** | **df** | **F** | **p-value** | **η2** |
| --- | --- | --- | --- | --- | --- | --- |
| **ASPL** | 3.167 | 3.305 | 1996 | 12.239 | <.001 | 0.006 |
| **CC** | 0.701 | 0.696 | 1996 | 9.242 | 0.002 | 0.005 |
| **Q** | 0.592 | 0.599 | 1996 | 0.515 | 0.019 | 0.003 |

**Table 2. T-test results of the 2nd test**

|  | **FT** | **VPT** | **df** | **F** | **p-value** | **η2** |
| --- | --- | --- | --- | --- | --- | --- |
| **ASPL** | 3.124 | 3.354 | 1996 | 34.024 | <.001 | 0.017 |
| **CC** | 0.703 | 0.694 | 1996 | 31.456 | <.001 | 0.016 |
| **Q** | 0.592 | 0.601 | 1996 | 8.618 | 0.003 | 0.004 |

**Table 3. T-test results of the 3rd test**

|  | **FT** | **VPT** | **df** | **F** | **p-value** | **η2** |
| --- | --- | --- | --- | --- | --- | --- |
| **ASPL** | 3.18 | 3.298 | 1996 | 8.462 | 0.004 | 0.004 |
| **CC** | 0.701 | 0.696 | 1996 | 10.403 | 0.001 | 0.005 |
| **Q** | 0.593 | 0.599 | 1996 | 4.167 | 0.041 | 0.002 |

**Table 4. T-test results of the 4th test**

|  | **FT** | **VPT** | **df** | **F** | **p-value** | **η2** |
| --- | --- | --- | --- | --- | --- | --- |
| **ASPL** | 3.176 | 3.307 | 1996 | 10.966 | 0.001 | 0.005 |
| **CC** | 0.701 | 0.696 | 1996 | 7.953 | 0.005 | 0.004 |
| **Q** | 0.594 | 0.601 | 1996 | 5.412 | 0.02 | 0.003 |

**Table 5. T-test results of the 5^th^ test**

|  | **FT** | **VPT** | **df** | **F** | **p-value** | **η2** |
| --- | --- | --- | --- | --- | --- | --- |
| **ASPL** | 3.165 | 3.318 | 1996 | 13.726 | <.001 | 0.007 |
| **CC** | 0.702 | 0.696 | 1996 | 14.930 | <.001 | 0.007 |
| **Q** | 0.592 | 0.6 | 1996 | 7.385 | 0.007 | 0.004 |

**Table 6. T-test results of the 6^th^ test**

|  | **FT** | **VPT** | **df** | **F** | **p-value** | **η2** |
| --- | --- | --- | --- | --- | --- | --- |
| **ASPL** | 3.141 | 3.331 | 1996 | 21.853 | <.001 | 0.011 |
| **CC** | 0.703 | 0.695 | 1996 | 19.355 | <.001 | 0.010 |
| **Q** | 0.592 | 0.6 | 1996 | 5.683 | 0.017 | 0.003 |

**Table 7. T-test results of the 7^th^ test**

|  | **FT** | **VPT** | **df** | **F** | **p-value** | **η2** |
| --- | --- | --- | --- | --- | --- | --- |
| **ASPL** | 3.161 | 3.324 | 1996 | 14.910 | <.001 | 0.007 |
| **CC** | 0.702 | 0.695 | 1996 | 16.038 | <.001 | 0.008 |
| **Q** | 0.592 | 0.602 | 1996 | 10.270 | 0.001 | 0.005 |

**Table 8. T-test results of the 8^th^ test**

|  | **FT** | **VPT** | **df** | **F** | **p-value** | **η2** |
| --- | --- | --- | --- | --- | --- | --- |
| **ASPL** | 3.103 | 3.374 | 1996 | 44.441 | <.001 | 0.022 |
| **CC** | 0.703 | 0.694 | 1996 | 27.562 | <.001 | 0.014 |
| **Q** | 0.589 | 0.603 | 1996 | 20.574 | <.001 | 0.010 |

**Table 9. T-test results of the 9^th^ test**

|  | **FT** | **VPT** | **df** | **F** | **p-value** | **η2** |
| --- | --- | --- | --- | --- | --- | --- |
| **ASPL** | 3.197 | 3.278 | 1996 | 4.439 | 0.035 | 0.002 |
| **CC** | 0.701 | 0.696 | 1996 | 8.502 | 0.004 | 0.004 |
| **Q** | 0.593 | 0.601 | 1996 | 7.430 | 0.006 | 0.004 |

**Table 10. T-test results of the 10^th^ test**

|  | **FT** | **VPT** | **df** | **F** | **p-value** | **η2** |
| --- | --- | --- | --- | --- | --- | --- |
| **ASPL** | 3.173 | 3.31 | 1996 | 11.627 | 0.001 | 0.006 |
| **CC** | 0.701 | 0.696 | 1996 | 8.648 | 0.003 | 0.004 |
| **Q** | 0.594 | 0.598 | 1996 | 1.730 | 0.189 | 0.001 |

**Table 11. T-test results of the 11^th^ test**

|  | **FT** | **VPT** | **df** | **F** | **p-value** | **η2** |
| --- | --- | --- | --- | --- | --- | --- |
| **ASPL** | 3.157 | 3.326 | 1996 | 17.042 | <.001 | 0.008 |
| **CC** | 0.702 | 0.695 | 1996 | 14.619 | <.001 | 0.007 |
| **Q** | 0.593 | 0.602 | 1996 | 8.354 | 0.004 | 0.004 |

**Table 12. T-test results of the 12^th^ test**

|  | **FT** | **VPT** | **df** | **F** | **p-value** | **η2** |
| --- | --- | --- | --- | --- | --- | --- |
| **ASPL** | 3.181 | 3.309 | 1996 | 9.854 | 0.002 | 0.005 |
| **CC** | 0.701 | 0.696 | 1996 | 9.678 | 0.002 | 0.005 |
| **Q** | 0.595 | 0.599 | 1996 | 1.943 | 0.164 | 0.001 |

**Table 13. T-test results of the 13^th^ test**

|  | **FT** | **VPT** | **df** | **F** | **p-value** | **η2** |
| --- | --- | --- | --- | --- | --- | --- |
| **ASPL** | 3.165 | 3.293 | 1996 | 10.769 | 0.001 | 0.005 |
| **CC** | 0.701 | 0.696 | 1996 | 10.096 | 0.002 | 0.005 |
| **Q** | 0.594 | 0.597 | 1996 | 1.040 | 0.308 | 0.001 |

**Table 14. T-test results of the 14^th^ test**

|  | **FT** | **VPT** | **df** | **F** | **p-value** | **η2** |
| --- | --- | --- | --- | --- | --- | --- |
| **ASPL** | 3.139 | 3.33 | 1996 | 22.404 | <.001 | 0.011 |
| **CC** | 0.702 | 0.695 | 1996 | 16.833 | <.001 | 0.008 |
| **Q** | 0.591 | 0.601 | 1996 | 11.267 | 0.001 | 0.006 |

**Table 15. T-test results of the 15^th^ test**

|  | **FT** | **VPT** | **df** | **F** | **p-value** | **η2** |
| --- | --- | --- | --- | --- | --- | --- |
| **ASPL** | 3.153 | 3.311 | 1996 | 15.990 | <.001 | 0.008 |
| **CC** | 0.702 | 0.696 | 1996 | 12.032 | 0.001 | 0.006 |
| **Q** | 0.592 | 0.598 | 1996 | 3.792 | 0.052 | 0.002 |

**Table 16. T-test results of the 16^th^ test**

|  | **FT** | **VPT** | **df** | **F** | **p-value** | **η2** |
| --- | --- | --- | --- | --- | --- | --- |
| **ASPL** | 3.152 | 3.348 | 1996 | 24.956 | <.001 | 0.012 |
| **CC** | 0.702 | 0.694 | 1996 | 21.011 | <.001 | 0.010 |
| **Q** | 0.59 | 0.602 | 1996 | 13.924 | <.001 | 0.007 |

**Table 17. T-test results of the 17^th^ test**

|  | **FT** | **VPT** | **df** | **F** | **p-value** | **η2** |
| --- | --- | --- | --- | --- | --- | --- |
| **ASPL** | 3.139 | 3.327 | 1996 | 21.690 | <.001 | 0.011 |
| **CC** | 0.702 | 0.695 | 1996 | 16.076 | <.001 | 0.008 |
| **Q** | 0.591 | 0.601 | 1996 | 11.431 | 0.001 | 0.006 |

**Table 18. T-test results of the 18^th^ test**

|  | **FT** | **VPT** | **df** | **F** | **p-value** | **η2** |
| --- | --- | --- | --- | --- | --- | --- |
| **ASPL** | 3.182 | 3.301 | 1996 | 8.607 | 0.003 | 0.004 |
| **CC** | 0.702 | 0.695 | 1996 | 16.433 | <.001 | 0.008 |
| **Q** | 0.593 | 0.599 | 1996 | 3.004 | 0.083 | 0.002 |

**Table 19. T-test results of the 19^th^ test**

|  | **FT** | **VPT** | **df** | **F** | **p-value** | **η2** |
| --- | --- | --- | --- | --- | --- | --- |
| **ASPL** | 3.168 | 3.323 | 1996 | 13.393 | <.001 | 0.007 |
| **CC** | 0.702 | 0.695 | 1996 | 14.087 | <.001 | 0.007 |
| **Q** | 0.594 | 0.6 | 1996 | 3.275 | 0.07 | 0.002 |

**Table 20. T-test results of the 20^th^ test**

|  | **FT** | **VPT** | **df** | **F** | **p-value** | **η2** |
| --- | --- | --- | --- | --- | --- | --- |
| **ASPL** | 3.137 | 3.34 | 1996 | 26.295 | <.001 | 0.013 |
| **CC** | 0.702 | 0.695 | 1996 | 18.473 | <.001 | 0.009 |
| **Q** | 0.592 | 0.601 | 1996 | 8.549 | 0.003 | 0.004 |

**Fig 2. Bar plot of the twenty bootstrap analyses of the unadjusted model**


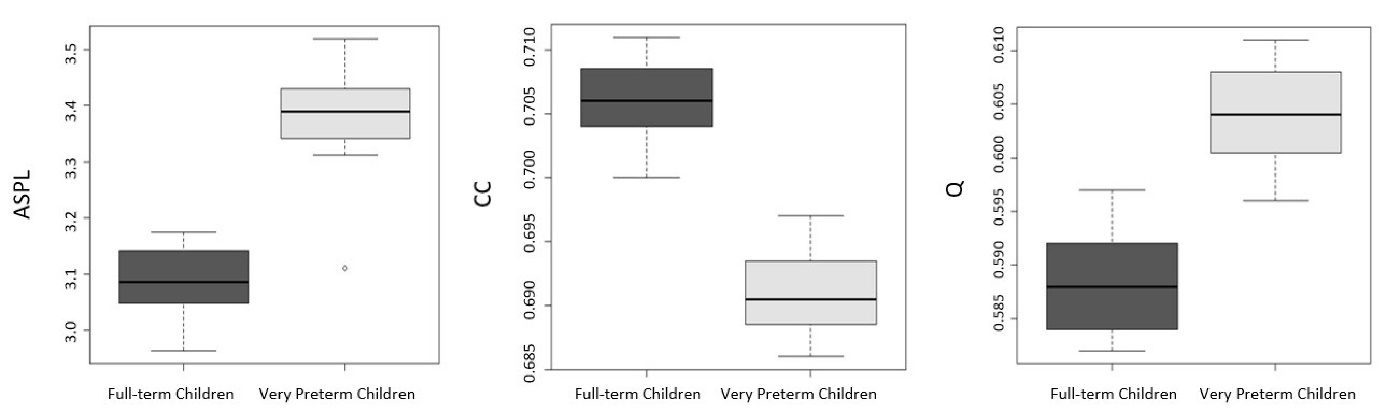


Average of the 20 case-wise bootstrap tests for the 3 coefficients separated by groups. *Note*. ASPL = Average Shortest Path Length, CC = Clustering Coefficient and Q = Modularity
