# Supplementary material for: Characterization of language abilities and semantic networks in very preterm children at school-age
Source: PLoS One. 2025 Jan 29;20(1):e0317535. doi: 10.1371/journal.pone.0317535 (PMC12140111; doi:10.1371/journal.pone.0317535)
Supplement: S2 File — (DOCX) [file pone.0317535.s003.docx]

**Adjusted model for the SES**

**Results of the twenty case-wise bootstrap network analyses**

**Table 21. ANCOVA results of the 1rst test**

|  | **FT** | **VPT** | **df** | **F** | **p-value** | **η2** |
| --- | --- | --- | --- | --- | --- | --- |
| **ASPL** | 3.188 | 3.308 | 1995 | 9.436 | 0.002 | 0.005 |
| **CC** | 0.701 | 0.695 | 1995 | 16.309 | <.001 | 0.008 |
| **Q** | 0.595 | 0.598 | 1995 | 1.077 | 0.3 | 0.001 |

**Table 22. ANCOVA results of the 2nd test**

|  | **FT** | **VPT** | **df** | **F** | **p-value** | **η2** |
| --- | --- | --- | --- | --- | --- | --- |
| **ASPL** | 3.2 | 3.285 | 1995 | 4.468 | 0.035 | 0.002 |
| **CC** | 0.701 | 0.696 | 1995 | 6.212 | 0.013 | 0.003 |
| **Q** | 0.597 | 0.597 | 1995 | 0.007 | 0.933 | 0.000 |

**Table 23. ANCOVA results of the 3rd test**

|  | **FT** | **VPT** | **df** | **F** | **p-value** | **η2** |
| --- | --- | --- | --- | --- | --- | --- |
| **ASPL** | 3.144 | 3.33 | 1995 | 21.181 | <.001 | 0.011 |
| **CC** | 0.702 | 0.695 | 1995 | 14.351 | <.001 | 0.007 |
| **Q** | 0.592 | 0.601 | 1995 | 7.177 | 0.007 | 0.004 |

**Table 24. ANCOVA results of the 4th test**

|  | **FT** | **VPT** | **df** | **F** | **p-value** | **η2** |
| --- | --- | --- | --- | --- | --- | --- |
| **ASPL** | 3.117 | 3.348 | 1995 | 34.595 | <.001 | 0.017 |
| **CC** | 0.703 | 0.695 | 1995 | 20.817 | <.001 | 0.010 |
| **Q** | 0.593 | 0.601 | 1995 | 7.696 | 0.006 | 0.004 |

**Table 25. ANCOVA results of the 5^th^ test**

|  | **FT** | **VPT** | **df** | **F** | **p-value** | **η2** |
| --- | --- | --- | --- | --- | --- | --- |
| **ASPL** | 3.169 | 3.334 | 1995 | 15.126 | <.001 | 0.008 |
| **CC** | 0.7 | 0.696 | 1995 | 4.975 | 0.026 | 0.002 |
| **Q** | 0.595 | 0.599 | 1995 | 2.101 | 0.147 | 0.001 |

**Table 26. ANCOVA results of the 6^th^ test**

|  | **FT** | **VPT** | **df** | **F** | **p-value** | **η2** |
| --- | --- | --- | --- | --- | --- | --- |
| **ASPL** | 3.143 | 3.354 | 1995 | 28.744 | <.001 | 0.014 |
| **CC** | 0.702 | 0.694 | 1995 | 23.916 | <.001 | 0.012 |
| **Q** | 0.591 | 0.602 | 1995 | 12.927 | <.001 | 0.006 |

**Table 27. ANCOVA results of the 7^th^ test**

|  | **FT** | **VPT** | **df** | **F** | **p-value** | **η2** |
| --- | --- | --- | --- | --- | --- | --- |
| **ASPL** | 3.135 | 3.313 | 1995 | 19.666 | <.001 | 0.010 |
| **CC** | 0.702 | 0.696 | 1995 | 15.373 | <.001 | 0.008 |
| **Q** | 0.59 | 0.6 | 1995 | 11.748 | 0.001 | 0.006 |

**Table 28. ANCOVA results of the 8^th^ test**

|  | **FT** | **VPT** | **df** | **F** | **p-value** | **η2** |
| --- | --- | --- | --- | --- | --- | --- |
| **ASPL** | 3.155 | 3.313 | 1995 | 16.634 | <.001 | 0.008 |
| **CC** | 0.701 | 0.696 | 1995 | 10.225 | 0.001 | 0.005 |
| **Q** | 0.594 | 0.6 | 1995 | 4.657 | 0.031 | 0.002 |

**Table 29. ANCOVA results of the 9^th^ test**

|  | **FT** | **VPT** | **df** | **F** | **p-value** | **η2** |
| --- | --- | --- | --- | --- | --- | --- |
| **ASPL** | 3.175 | 3.33 | 1995 | 14.953 | <.001 | 0.007 |
| **CC** | 0.702 | 0.695 | 1995 | 17.673 | <.001 | 0.009 |
| **Q** | 0.593 | 0.6 | 1995 | 4.709 | 0.03 | 0.002 |

**Table 30. ANCOVA results of the 10^th^ test**

|  | **FT** | **VPT** | **df** | **F** | **p-value** | **η2** |
| --- | --- | --- | --- | --- | --- | --- |
| **ASPL** | 3.163 | 3.324 | 1995 | 15.936 | <.001 | 0.008 |
| **CC** | 0.701 | 0.696 | 1995 | 10.884 | 0.001 | 0.005 |
| **Q** | 0.592 | 0.601 | 1995 | 9.951 | 0.002 | 0.005 |

**Table 31. ANCOVA results of the 11^th^ test**

|  | **FT** | **VPT** | **df** | **F** | **p-value** | **η2** |
| --- | --- | --- | --- | --- | --- | --- |
| **ASPL** | 3.192 | 3.289 | 1995 | 5.977 | 0.015 | 0.003 |
| **CC** | 0.703 | 0.695 | 1995 | 17.488 | <.001 | 0.009 |
| **Q** | 0.589 | 0.604 | 1995 | 22.780 | <.001 | 0.011 |

**Table 32. ANCOVA results of the 12^th^ test**

|  | **FT** | **VPT** | **df** | **F** | **p-value** | **η2** |
| --- | --- | --- | --- | --- | --- | --- |
| **ASPL** | 3.168 | 3.32 | 1995 | 15.055 | <.001 | 0.007 |
| **CC** | 0.701 | 0.695 | 1995 | 13.822 | <.001 | 0.007 |
| **Q** | 0.594 | 0.6 | 1995 | 3.417 | 0.065 | 0.002 |

**Table 33. ANCOVA results of the 13^th^ test**

|  | **FT** | **VPT** | **df** | **F** | **p-value** | **η2** |
| --- | --- | --- | --- | --- | --- | --- |
| **ASPL** | 3.2 | 3.298 | 1995 | 6.255 | 0.012 | 0.003 |
| **CC** | 0.702 | 0.695 | 1995 | 14.545 | <.001 | 0.007 |
| **Q** | 0.594 | 0.6 | 1995 | 4.172 | 0.041 | 0.002 |

**Table 34. ANCOVA results of the 14^th^ test**

|  | **FT** | **VPT** | **df** | **F** | **p-value** | **η2** |
| --- | --- | --- | --- | --- | --- | --- |
| **ASPL** | 3.153 | 3.325 | 1995 | 17.808 | <.001 | 0.009 |
| **CC** | 0.703 | 0.694 | 1995 | 28.119 | <.001 | 0.014 |
| **Q** | 0.594 | 0.6 | 1995 | 3.756 | 0.053 | 0.002 |

**Table 35. ANCOVA results of the 15^th^ test**

|  | **FT** | **VPT** | **df** | **F** | **p-value** | **η2** |
| --- | --- | --- | --- | --- | --- | --- |
| **ASPL** | 3.158 | 3.298 | 1995 | 12.040 | 0.001 | 0.006 |
| **CC** | 0.701 | 0.696 | 1995 | 8.551 | 0.003 | 0.004 |
| **Q** | 0.593 | 0.599 | 1995 | 4.013 | 0.045 | 0.002 |

**Table 36. ANCOVA results of the 16^th^ test**

|  | **FT** | **VPT** | **df** | **F** | **p-value** | **η2** |
| --- | --- | --- | --- | --- | --- | --- |
| **ASPL** | 3.187 | 3.289 | 1995 | 6.572 | 0.01 | 0.003 |
| **CC** | 0.701 | 0.697 | 1995 | 3.747 | 0.053 | 0.002 |
| **Q** | 0.594 | 0.597 | 1995 | 1.355 | 0.245 | 0.001 |

**Table 37. ANCOVA results of the 17^th^ test**

|  | **FT** | **VPT** | **df** | **F** | **p-value** | **η2** |
| --- | --- | --- | --- | --- | --- | --- |
| **ASPL** | 3.163 | 3.311 | 1995 | 14.218 | <.001 | 0.007 |
| **CC** | 0.702 | 0.695 | 1995 | 14.791 | <.001 | 0.007 |
| **Q** | 0.594 | 0.598 | 1995 | 2.411 | 0.121 | 0.001 |

**Table 38. ANCOVA results of the 18^th^ test**

|  | **FT** | **VPT** | **df** | **F** | **p-value** | **η2** |
| --- | --- | --- | --- | --- | --- | --- |
| **ASPL** | 3.168 | 3.283 | 1995 | 8.978 | 0.003 | 0.004 |
| **CC** | 0.702 | 0.696 | 1995 | 10.049 | 0.002 | 0.005 |
| **Q** | 0.594 | 0.597 | 1995 | 1.200 | 0.273 | 0.001 |

**Table 39. ANCOVA results of the 19^th^ test**

|  | **FT** | **VPT** | **df** | **F** | **p-value** | **η2** |
| --- | --- | --- | --- | --- | --- | --- |
| **ASPL** | 3.21 | 3.281 | 1995 | 2.962 | 0.085 | 0.001 |
| **CC** | 0.7 | 0.696 | 1995 | 4.834 | 0.028 | 0.002 |
| **Q** | 0.595 | 0.598 | 1995 | 1.396 | 0.238 | 0.001 |

**Table 40. ANCOVA results of the 20^th^ test**

|  | **FT** | **VPT** | **df** | **F** | **p-value** | **η2** |
| --- | --- | --- | --- | --- | --- | --- |
| **ASPL** | 3.174 | 3.304 | 1995 | 10.262 | 0.001 | 0.005 |
| **CC** | 0.701 | 0.696 | 1995 | 9.121 | 0.003 | 0.005 |
| **Q** | 0.592 | 0.601 | 1995 | 9.948 | 0.002 | 0.005 |

**Fig 3. Bar plot of the twenty bootstrap analyses of the adjusted model with SES**


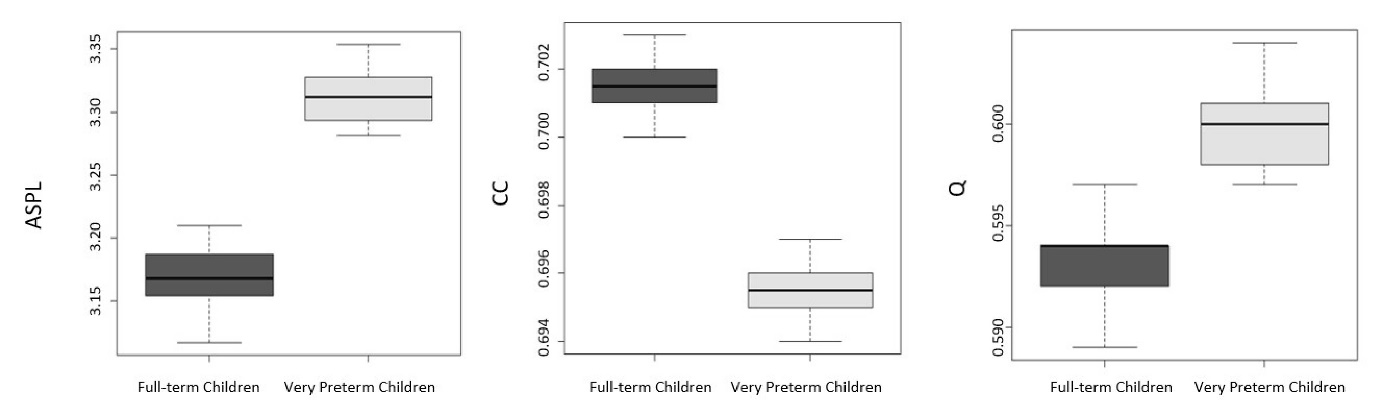


Average of the 20 case-wise bootstrap tests with SES as a covariate for the 3 coefficients separated by groups.
