# Supplementary material for: Characterization of language abilities and semantic networks in very preterm children at school-age
Source: PLoS One. 2025 Jan 29;20(1):e0317535. doi: 10.1371/journal.pone.0317535 (PMC12140111; doi:10.1371/journal.pone.0317535)
Supplement: S3 File — (DOCX) [file pone.0317535.s004.docx]

**Adjusted model for the SES and Intelligence**

**Results of the twenty case-wise bootstrap network analyses**

**Table 41. ANCOVA results of the 1rst test**

|  | **FT** | **VPT** | **df** | **F** | **p-value** | **η^2^** |
| --- | --- | --- | --- | --- | --- | --- |
| **ASPL** | 3.073 | 3.404 | 1994 | 7.454 | 0.006 | 0.004 |
| **CC** | 0.711 | 0.687 | 1994 | 20.966 | <.001 | 0.010 |
| **Q** | 0.585 | 0.607 | 1994 | 5.626 | 0.018 | 0.003 |

**Table 42. ANCOVA results of the 2nd test**

|  | **FT** | **VPT** | **df** | **F** | **p-value** | **η^2^** |
| --- | --- | --- | --- | --- | --- | --- |
| **ASPL** | 3.143 | 3.342 | 1994 | 2.418 | 0.12 | 0.001 |
| **CC** | 0.705 | 0.692 | 1994 | 4.945 | 0.026 | 0.002 |
| **Q** | 0.591 | 0.602 | 1994 | 1.416 | 0.234 | 0.001 |

**Table 43. ANCOVA results of the 3rd test**

|  | **FT** | **VPT** | **df** | **F** | **p-value** | **η^2^** |
| --- | --- | --- | --- | --- | --- | --- |
| **ASPL** | 3.139 | 3.343 | 1994 | 2.737 | 0.098 | 0.001 |
| **CC** | 0.704 | 0.693 | 1994 | 4.710 | 0.03 | 0.002 |
| **Q** | 0.593 | 0.6 | 1994 | 0.665 | 0.415 | 0.000 |

**Table 44. ANCOVA results of the 4th test**

|  | **FT** | **VPT** | **df** | **F** | **p-value** | **η^2^** |
| --- | --- | --- | --- | --- | --- | --- |
| **ASPL** | 3.035 | 3.449 | 1994 | 10.898 | 0.001 | 0.005 |
| **CC** | 0.707 | 0.69 | 1994 | 9.327 | 0.002 | 0.005 |
| **Q** | 0.588 | 0.604 | 1994 | 2.747 | 0.098 | 0.001 |

**Table 45. ANCOVA results of the 5^th^ test**

|  | **FT** | **VPT** | **df** | **F** | **p-value** | **η^2^** |
| --- | --- | --- | --- | --- | --- | --- |
| **ASPL** | 3.141 | 3.34 | 1994 | 2.561 | 0.11 | 0.001 |
| **CC** | 0.703 | 0.694 | 1994 | 3.157 | 0.076 | 0.002 |
| **Q** | 0.597 | 0.596 | 1994 | 0.006 | 0.937 | 0.000 |

**Table 46. ANCOVA results of the 6^th^ test**

|  | **FT** | **VPT** | **df** | **F** | **p-value** | **η^2^** |
| --- | --- | --- | --- | --- | --- | --- |
| **ASPL** | 3.063 | 3.393 | 1994 | 7.308 | 0.007 | 0.004 |
| **CC** | 0.704 | 0.694 | 1994 | 3.446 | 0.064 | 0.002 |
| **Q** | 0.582 | 0.609 | 1994 | 8.307 | 0.004 | 0.004 |

**Table 47. ANCOVA results of the 7^th^ test**

|  | **FT** | **VPT** | **df** | **F** | **p-value** | **η^2^** |
| --- | --- | --- | --- | --- | --- | --- |
| **ASPL** | 3.05 | 3.415 | 1994 | 8.676 | 0.003 | 0.004 |
| **CC** | 0.71 | 0.688 | 1994 | 17.297 | <.001 | 0.009 |
| **Q** | 0.582 | 0.609 | 1994 | 8.143 | 0.004 | 0.004 |

**Table 48. ANCOVA results of the 8^th^ test**

|  | **FT** | **VPT** | **df** | **F** | **p-value** | **η^2^** |
| --- | --- | --- | --- | --- | --- | --- |
| **ASPL** | 3.165 | 3.11 | 1994 | 1.267 | 0.261 | 0.001 |
| **CC** | 0.7 | 0.697 | 1994 | 0.199 | 0.655 | 0.000 |
| **Q** | 0.593 | 0.599 | 1994 | 0.446 | 0.505 | 0.000 |

**Table 49. ANCOVA results of the 9^th^ test**

|  | **FT** | **VPT** | **df** | **F** | **p-value** | **η^2^** |
| --- | --- | --- | --- | --- | --- | --- |
| **ASPL** | 3.174 | 3.312 | 1994 | 1.127 | 0.289 | 0.001 |
| **CC** | 0.7 | 0.697 | 1994 | 0.279 | 0.598 | 0.000 |
| **Q** | 0.596 | 0.596 | 1994 | 0.000 | 0.989 | 0.000 |

**Table 50. ANCOVA results of the 10^th^ test**

|  | **FT** | **VPT** | **df** | **F** | **p-value** | **η^2^** |
| --- | --- | --- | --- | --- | --- | --- |
| **ASPL** | 2.994 | 3.467 | 1994 | 14.039 | <.001 | 0.007 |
| **CC** | 0.709 | 0.688 | 1994 | 13.832 | <.001 | 0.007 |
| **Q** | 0.582 | 0.609 | 1994 | 8.046 | 0.005 | 0.004 |

**Table 51. ANCOVA results of the 11^th^ test**

|  | **FT** | **VPT** | **df** | **F** | **p-value** | **η^2^** |
| --- | --- | --- | --- | --- | --- | --- |
| **ASPL** | 3.159 | 3.323 | 1994 | 1.742 | 0.187 | 0.001 |
| **CC** | 0.704 | 0.693 | 1994 | 3.986 | 0.046 | 0.002 |
| **Q** | 0.596 | 0.597 | 1994 | 0.016 | 0.9 | 0.000 |

**Table 52. ANCOVA results of the 12^th^ test**

|  | **FT** | **VPT** | **df** | **F** | **p-value** | **η^2^** |
| --- | --- | --- | --- | --- | --- | --- |
| **ASPL** | 3.157 | 3.323 | 1994 | 1.660 | 0.198 | 0.001 |
| **CC** | 0.7 | 0.697 | 1994 | 0.238 | 0.626 | 0.000 |
| **Q** | 0.59 | 0.601 | 1994 | 1.268 | 0.26 | 0.001 |

**Table 53. ANCOVA results of the 13^th^ test**

|  | **FT** | **VPT** | **df** | **F** | **p-value** | **η^2^** |
| --- | --- | --- | --- | --- | --- | --- |
| **ASPL** | 3.075 | 3.393 | 1994 | 6.580 | 0.01 | 0.003 |
| **CC** | 0.704 | 0.693 | 1994 | 3.750 | 0.053 | 0.002 |
| **Q** | 0.588 | 0.604 | 1994 | 2.571 | 0.109 | 0.001 |

**Table 54. ANCOVA results of the 14^th^ test**

|  | **FT** | **VPT** | **df** | **F** | **p-value** | **η^2^** |
| --- | --- | --- | --- | --- | --- | --- |
| **ASPL** | 3.052 | 3.43 | 1994 | 8.515 | 0.004 | 0.004 |
| **CC** | 0.708 | 0.689 | 1994 | 12.461 | <.001 | 0.006 |
| **Q** | 0.589 | 0.603 | 1994 | 2.394 | 0.122 | 0.001 |

**Table 55. ANCOVA results of the 15^th^ test**

|  | **FT** | **VPT** | **df** | **F** | **p-value** | **η^2^** |
| --- | --- | --- | --- | --- | --- | --- |
| **ASPL** | 3.137 | 3.371 | 1994 | 3.317 | 0.069 | 0.002 |
| **CC** | 0.706 | 0.69 | 1994 | 8.696 | 0.003 | 0.004 |
| **Q** | 0.583 | 0.611 | 1994 | 9.365 | 0.002 | 0.005 |

**Table 56. ANCOVA results of the 16^th^ test**

|  | **FT** | **VPT** | **df** | **F** | **p-value** | **η^2^** |
| --- | --- | --- | --- | --- | --- | --- |
| **ASPL** | 3.044 | 3.431 | 1994 | 9.674 | 0.002 | 0.005 |
| **CC** | 0.707 | 0.69 | 1994 | 10.442 | 0.001 | 0.005 |
| **Q** | 0.584 | 0.608 | 1994 | 6.158 | 0.011 | 0.003 |

**Table 57. ANCOVA results of the 17^th^ test**

|  | **FT** | **VPT** | **df** | **F** | **p-value** | **η^2^** |
| --- | --- | --- | --- | --- | --- | --- |
| **ASPL** | 3.121 | 3.343 | 1994 | 3.236 | 0.072 | 0.002 |
| **CC** | 0.706 | 0.691 | 1994 | 7.184 | 0.007 | 0.004 |
| **Q** | 0.589 | 0.602 | 1994 | 2.139 | 0.144 | 0.001 |

**Table 58. ANCOVA results of the 18^th^ test**

|  | **FT** | **VPT** | **df** | **F** | **p-value** | **η^2^** |
| --- | --- | --- | --- | --- | --- | --- |
| **ASPL** | 2.963 | 3.519 | 1994 | 20.212 | <.001 | 0.010 |
| **CC** | 0.711 | 0.686 | 1994 | 20.619 | <.001 | 0.010 |
| **Q** | 0.587 | 0.605 | 1994 | 3.465 | 0.063 | 0.002 |

**Table 59. ANCOVA results of the 19^th^ test**

|  | **FT** | **VPT** | **df** | **F** | **p-value** | **η^2^** |
| --- | --- | --- | --- | --- | --- | --- |
| **ASPL** | 3.021 | 3.474 | 1994 | 12.216 | <.001 | 0.006 |
| **CC** | 0.71 | 0.687 | 1994 | 17.463 | <.001 | 0.009 |
| **Q** | 0.586 | 0.607 | 1994 | 4.548 | 0.033 | 0.002 |

**Table 60. ANCOVA results of the 20^th^ test**

|  | **FT** | **VPT** | **df** | **F** | **p-value** | **η^2^** |
| --- | --- | --- | --- | --- | --- | --- |
| **ASPL** | 3.097 | 3.385 | 1994 | 5.096 | 0.024 | 0.003 |
| **CC** | 0.707 | 0.69 | 1994 | 10.107 | 0.001 | 0.005 |
| **Q** | 0.584 | 0.608 | 1994 | 6.593 | 0.01 | 0.003 |

**Fig 4. Bar plot of the twenty bootstrap analyses of the model adjusted for SES and non-verbal intelligence**


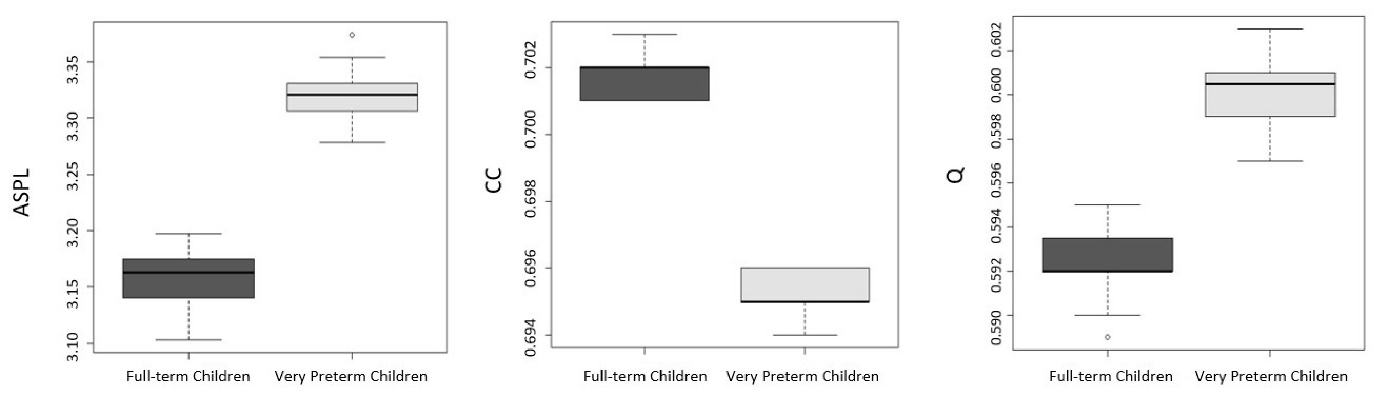


Average of the 20 case-wise bootstrap tests with SES and non-verbal intelligence as covariates for the 3 coefficients separated by groups.
